# Spotlight on the treatment of *ALK*-rearranged non-small-cell lung cancer

**DOI:** 10.2217/lmt-2018-0004

**Published:** 2018-06-22

**Authors:** Hirva Mamdani, Shadia I Jalal

**Affiliations:** 1 Department of Hematology & Oncology, Karmanos Cancer Institute/Wayne State University, Detroit, MI, USA; 2 Department of Medicine, Indiana University, Melvin & Bren Simon Cancer Center, Division of Hematology/Oncology, Indianapolis, IN, USA

**Keywords:** alectinib, *ALK* rearrangement, brigatinib, ceritinib, crizotinib, molecularly targeted therapy, non-small-cell lung cancer

Lung cancer is the leading cause of cancer-related mortality, both worldwide and in the USA. Non-small-cell lung cancer (NSCLC) accounts for approximately 85% of all lung cancer cases. At the turn of 21st century, platinum based cytotoxic chemotherapy was shown to offer modest survival benefit in metastatic NSCLC and remained the only viable treatment option for a long time. Over the past decade, the therapeutic landscape of NSCLC has expanded dramatically owing to the discovery of various driver mutations. Several molecularly targeted agents and immune checkpoint inhibitors are now a part of the therapeutic armamentarium against this genetically complex disease.

*ALK* gene encodes for a member of insulin receptor superfamily transmembrane receptor tyrosine kinase [1]. In 2007, chromosomal rearrangement involving *ALK* gene on chromosome 2 and *EML4* gene on chromosome 5 was first found to have potent transforming activity in NSCLC. Subsequently, preclinical studies suggested that this fusion gene might be the driver mutation and potentially be a therapeutic target of NSCLC [2]. Approximately, 3–7% of patients with NSCLC harbor the *EML4–ALK* gene rearrangement, which is mutually exclusive with *EGFR* and *KRAS* mutations. *ALK* gene rearrangements are more common in younger patients with adenocarcinoma histology and those with minimal or no smoking history. There are reports of *ALK* gene rearrangement in patients with squamous cell and small-cell lung cancer; however, its clinical significance and potential as a therapeutic target in these histologic subtypes remain unknown. The testing modalities for *ALK* rearrangement in NSCLC include immunohistochemistry (IHC), FISH, and PCR; with the former two being the most commonly utilized modalities. However, there is a variable rate of discordance in response to *ALK* inhibition in IHC-negative but FISH-positive tumors, and therefore both IHC and FISH are currently recommended for *ALK* testing.

Crizotinib, originally developed as a *c-MET* inhibitor, is the first-in-class *ALK* inhibitor to show activity in *ALK*-rearranged NSCLC. In addition, it is also active in *ROS1*-rearranged lung cancer. Crizotinib received accelerated US FDA approval in 2011 based on a Phase I trial showing objective response rate (ORR) of 60% with a median progression free survival (PFS) of 9.7 months and 12-month overall survival of 74.8% in patients with *ALK*-rearranged NSCLC [3]. Subsequently, two randomized Phase III trials comparing crizotinib with standard chemotherapy in second line and first-line settings confirmed significantly higher response rates and longer PFS with crizotinib. No statistically significant overall survival difference was observed in either of these trials, largely accounted for by significant crossover between the two arms [4,5].

Despite the striking results with this first *ALK* inhibitor, the success in personalized therapy was fraught with several challenges. First, the majority of patients develop resistance to crizotinib within the first 12 months of treatment. Several resistance mechanisms have been implicated and are broadly divided into two categories: *ALK*-dominant, and *ALK*-nondominant [6]. *ALK*-dominant mechanisms predominantly comprise second mutations in the *ALK* gene which include a gatekeeper mutation L1196M as well as other more recently reported mutations such as C1156Y, L1152R, 1151Tins, G1202R, S1206Y, F1174C, D1203N and G1269A [7]. *ALK*-nondominant mechanisms involve mutations in other oncogenes such as *EGFR* and *KRAS*, amplification of *KIT*, and transformation to sarcomatoid carcinoma [8]. Second, CNS penetration of crizotinib is inadequate leading to disease progression in CNS even in the presence of continued systemic response.

To overcome these challenges, a number of more potent and more selective *ALK* inhibitors have been developed. Among these, second generation *ALK* inhibitors ceritinib, alectinib and brigatinib are currently approved by the FDA. Ceritinib has shown activity in both, crizotinib naive and crizotinib resistant NSCLC, with improvement in ORR and PFS leading to its approval as a second-line agent and subsequently first-line agent in patients with advanced *ALK*-rearranged NSCLC [9–11]. Additionally, ceritinib has demonstrated superior CNS activity with intracranial response rate of 57% compared with 22% with crizotinib [12].

Alectinib is ten-times more potent *ALK*-inhibitor than crizotinib and has also shown efficacy in both, crizotinib naive and crizotinib resistant *ALK*-rearranged NSCLC, including the ones with gatekeeper mutation L1196M. Early phase trials of alectinib demonstrated its excellent efficacy in previously untreated *ALK*-rearranged NSCLC patients with ORR as high as 93.5% and more strikingly, no progression in the CNS in all enrolled patients [13,14]. Two randomized Phase III studies, J-ALEX and ALEX, comparing alectinib with crizotinib in previously untreated patients with *ALK*-positive advanced NSCLC showed higher response rate, significantly prolonged event-free survival, and lower rate of CNS progression with alectinib. In addition, alectinib was associated with lower rates of grade 3–5 adverse events. These trials have led to approval of alectinib as a first line agent in the treatment of *ALK*-positive NSCLC [15,16].

Alectinib offers substantial advantages over crizotinib in several aspects. First, it is active in *ALK*-positive NSCLC resistant to crizotinib mediated by L1196M gatekeeper mutation, thereby conferring continued benefit in patients who have disease progression while on crizotinib. Second, alectinib has significantly higher bioavailability in CNS and can produce rapid and durable response in patients with brain metastases [17]. In addition, a recent report suggested that in cases of disease progression in CNS after initial response to alectinib, dose intensification from 600 to 900 mg twice daily can produce another durable response in CNS, particularly in patients with leptomeningeal carcinomatosis [18]. Finally, alectinib has shown potent antitumor activity against *RET*-rearranged NSCLC indicating its potential role in this subset of patients. In contrast to crizotinib, alectinib does not have any appreciable activity against *MET* amplification or *ROS1* rearrangement.

Brigatinib is another potent *ALK* inhibitor with an ability to overcome crizotinib resistance mutations [19]. Similar to alectinib, brigatinib yields high response rates, prolongs PFS, and has good CNS activity [20]. Brigatinib is currently approved for the treatment of *ALK*-positive NSCLC following disease progression on crizotinib. In contrast to alectinib, it is active against *ALK* resistance mutation G1202R, *ROS1* and mutant *EGFR* including T790M. The three second-generation *ALK* inhibitors have not been compared head-to-head, but they differ in their efficacy and safety profiles as inferred from cross-trial comparisons. The efficacy appears to be lower with ceritinib as compared with alectinib or brigatinib. Alectinib has the most robust CNS activity data and is active against leptomeningeal disease as well [21]. With regards to safety profile, alectinib is best tolerated of the three *ALK* TKIs and dose reductions are rarely necessary. Ceritinib is associated with GI side effects that require frequent dose reductions. Finally, brigatinib is generally well tolerated; however, it is uniquely associated with pulmonary toxicity. Therefore, it needs to be started at a lead-in dose of 90 mg daily for 7 days, and then escalated to standard dose of 180 mg daily to minimize the risk of pulmonary toxicity.

Several third generation *ALK* inhibitors, including ensartinib, entrectinib and lorlatinib, are being studied in clinical trials [22,23]. Lorlatinib has obtained breakthrough therapy designation by the FDA and is currently available for previously treated *ALK*- and *ROS1*-positive NSCLC patients through an expanded access protocol. It is active in tumors harboring G1202R mutation in *ALK* that confers resistance to all first- and second-generation *ALK* inhibitors.

As with other TKIs in cancer treatment, almost all the patients eventually develop resistance to *ALK* inhibitors. The best strategy in that case is to obtain a biopsy of the growing lesion to analyze for the presence of resistance mutations in *ALK* and utilize another *ALK* inhibitor that is known to have efficacy against identified resistance mutation. There has been a case report of emergence of resistance mutations after disease progression on loralitnib that resensitized the tumor to crizotinib [24]. This strategy allows sequential use of various *ALK* inhibitors with continued benefit for several years. However, at some point in time, all tumors develop *ALK*-independent mechanisms of resistance, thereby requiring other treatment modalities.

The efficacy of immune checkpoint inhibitors after disease progression on TKIs in *ALK*-translocated NSCLC remains controversial. The majority of untreated *ALK*-translocated tumors demonstrate <50% PD-L1 expression. Moreover, more than 70% of previously PD-L1-negative tumors remain PD-L1-negative, and approximately 30% of previously PD-L1-positive tumors become negative after treatment with *ALK* inhibitors. Large randomized trials comparing immune checkpoint inhibitors to second-line chemotherapy included very few *ALK*-positive patients, however, the ORR and PFS with immune checkpoint inhibitors in this population were disappointing [25]. Therefore, current consensus is to utilize chemotherapy in patients with disease progression on *ALK* inhibitors. A number of clinical trials evaluating combination of *ALK* inhibitors with other treatment modalities such as the antiangiogenic agents and immune checkpoint inhibitors are currently ongoing. An interesting target in this realm is heat shock protein HSP90, a molecular chaperone that plays a central role in regulating the correct folding, stability and function of numerous proteins including EML4–ALK fusion protein [26]. Targeting the chaperone function of HSP90 is therefore an alternative approach to direct kinase inhibition for therapeutic intervention in *ALK*-driven NSCLC.

In summary, the treatment horizon of *ALK*-positive NSCLC has evolved by the discovery of a number of TKIs that target *ALK* gene rearrangement. The second generation *ALK* inhibitors have replaced crizotinib which once was the first line agent in the treatment of *ALK*-positive NSCLC. Two major advantages of newer *ALK* inhibitors over crizotinib include superior bioavailability in the CNS thereby conferring high response rates in CNS disease (both brain as well as leptomeningeal disease), and activity in the presence of certain resistance mutations. At present, it is unclear if other *ALK* inhibitors will yield appreciable and durable responses in the second line setting after alectinib; however, sequential treatment with *ALK* inhibitors selected based on the presence of resistance mutation can lead to continued disease response for several years. Eventual development of *ALK*-independent resistance mechanisms remains a challenge but the rapidly evolving field of personalized therapy will probably overcome this challenge in the future.
